# A comprehensive investigation of morphological features responsible for cerebral aneurysm rupture using machine learning

**DOI:** 10.1038/s41598-024-66840-1

**Published:** 2024-07-09

**Authors:** Mostafa Zakeri, Amirhossein Atef, Mohammad Aziznia, Azadeh Jafari

**Affiliations:** 1 CNNFM Lab, School of Mechanical Engineering, College of Engineering, University of Tehran, 1450 Kargar St. N., Tehran, 14399-57131 Iran; 2 Present Address: STRETCH Lab, Department of Biomedical Engineering and Mechanics, Virginia Tech, 330A Kelly Hall, 325 Stanger Street, Blacksburg, VA 24061 USA

**Keywords:** Cerebral aneurysm, Rupture prediction, Morphological parameters, Machine learning, Classification, Biomedical engineering, Applied mathematics, Computational models, Machine learning, Predictive medicine

## Abstract

Cerebral aneurysms are a silent yet prevalent condition that affects a significant global population. Their development can be attributed to various factors, presentations, and treatment approaches. The importance of selecting the appropriate treatment becomes evident upon diagnosis, as the severity of the disease guides the course of action. Cerebral aneurysms are particularly vulnerable in the circle of Willis and pose a significant concern due to the potential for rupture, which can lead to irreversible consequences, including fatality. The primary objective of this study is to predict the rupture status of cerebral aneurysms. To achieve this, we leverage a comprehensive dataset that incorporates clinical and morphological data extracted from 3D real geometries of previous patients. The aim of this research is to provide valuable insights that can help make informed decisions during the treatment process and potentially save the lives of future patients. Diagnosing and predicting aneurysm rupture based solely on brain scans is a significant challenge with limited reliability, even for experienced physicians. However, by employing statistical methods and machine learning techniques, we can assist physicians in making more confident predictions regarding rupture likelihood and selecting appropriate treatment strategies. To achieve this, we used 5 classification machine learning algorithms and trained them on a substantial database comprising 708 cerebral aneurysms. The dataset comprised 3 clinical features and 35 morphological parameters, including 8 novel morphological features introduced for the first time in this study. Our models demonstrated exceptional performance in predicting cerebral aneurysm rupture, with accuracy ranging from 0.76 to 0.82 and precision score from 0.79 to 0.83 for the test dataset. As the data are sensitive and the condition is critical, recall is prioritized as the more crucial parameter over accuracy and precision, and our models achieved outstanding recall score ranging from 0.85 to 0.92. Overall, the best model was Support Vector Machin with an accuracy and precision of 0.82, recall of 0.92 for the testing dataset and the area under curve of 0.84. The ellipticity index, size ratio, and shape irregularity are pivotal features in predicting aneurysm rupture, respectively, contributing significantly to our understanding of this complex condition. Among the multitude of parameters under investigation, these are particularly important. In this study, the ideal roundness parameter was introduced as a novel consideration and ranked fifth among all 38 parameters. Neck circumference and outlet numbers from the new parameters were also deemed significant contributors.

## Introduction

Cerebral aneurysms are prevalent brain disorders that are often asymptotic. They primarily manifest within the cerebral arterial circle, which is known as the Circle of Willis^1^. It is estimated that approximately 2%-5% of the world’s population has cerebral aneurysms^2^. The risk associated with aneurysms generally escalates with their size^3^. Various imaging techniques are used to diagnose aneurysms, including MRI, MRA, CTA, and CT, each with its own advantages and disadvantages^4^. Treatment options, such as clipping and stent placement, can reduce the rupture risk ^5^.

The rupture of aneurysms can be caused by various factors, including biological influences, such as the degradation of the vessel wall^6^, hemodynamic features, such as wall shear stress^7^, and clinical factors, such as smoking, obesity, diabetes, and a sedentary lifestyle^8^. Moreover, the location of the aneurysm is also considered an important parameter^9^. Geometric properties of an aneurysm are also significant indicators. An aspect ratio exceeding 1.6^10^ or a diameter-to-parent-vessel ratio greater than 2.3 render aneurysms more susceptible to rupture^11^. In a separate study, the surface ratio nonsphericity index was identified as a crucial parameter for predicting rupture up to two years in advance^12^. Zhang et al.^13,14^ identified irregular shape, larger size, and higher aspect ratio as important parameters for rupture risk. In addition, the surface area of the saccular section^15^, bifurcation angle^16^, and angle of the upcomping stream^17^ have been linked to the risk of rupture. Mocco et al.^18^ found that the perpendicular height and size ratio can also affect the rupture risk of cerebral aneurysms. The size and aspect ratios have been identified as important parameters in some studies^19–21^.

Several studies have used machine learning (ML) techniques to predict the rupture status or incidence of cerebral aneurysms. For instance, Heo et al. investigated the incidence of aneurysms among patients who had experienced a stroke, considering five risk groups^22^. Tanioka et al. achieved a commendable accuracy of 0.77 using morphological features based on data from 226 patients^23^. Ou et al. investigated 374 aneurysms and demonstrated that modern machine learning techniques outperform conventional logistic regression models^24^. A study of 1631 patients revealed that the best model for cerebral aneurysm rupture assessment was MLP, with an accuracy score of 0.79 and an AUC of 0.83^25^. However, it is crucial to recognize that cerebral aneurysm data are inherently sensitive, and metrics such as recall and precision are equally, if not more, important than accuracy^26^. In this context, the information of 124 patients was analyzed, and remarkable results were achieved with a precision of 0.8 and a recall score of 0.826 in predicting cerebral aneurysm rupture^27^. The inclusion of all available parameters in feature selection significantly enhanced model performance, with accuracy gains ranging from 0.10 to 0.35 when compared to considering only the five dominant parameters^28^. Apart from machine learning methods, some researchers have endeavored to develop novel approaches for predicting rupture risk. Lie et al., for instance, focused on constructing an institutional nomogram for this purpose^29^.

Our study addresses a critical gap in the current research on cerebral aneurysm rupture prediction. While previous investigations have explored various risk factors using traditional methods like linear regression, our work represents a significant advance by conducting a comprehensive analysis that integrates both established and novel morphological parameters. By evaluating 35 distinct features, including eight newly introduced parameters alongside three clinical metrics, we provide a more comprehensive understanding of rupture risk factors.

The novelty of our approach lies in the comprehensive and thorough nature of our analysis. Our approach goes beyond conventional methods by harnessing the power of five advanced machine learning models to develop a rapid and precise predictive method for cerebral aneurysm rupture. Our emphasis on not just accuracy but also precision and recall metrics allows for a detailed evaluation of feature importance. This methodology enables us to uncover the most influential factors contributing to aneurysm rupture, providing valuable insights for clinical practice and future research in this domain.

## Methodology

This section provides an overview of the dataset used, followed by a detailed examination of each morphological feature. The representation of each feature will be elucidated by considering both previously established and novel parameters. Finally, the selected machine learning algorithms are discussed, and the data preprocessing steps essential to render the data suitable as inputs for the models are described.

### Study population

**Figure 1:**
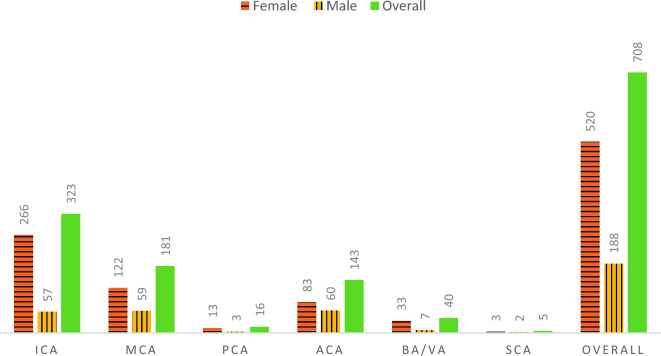
Location of aneurysms and patient’s gender.

The dataset used in this study was graciously provided by the AneuX morphology database, an open-access, multi-centric database combining data from three European projects: AneuX project (www.aneux.ch), @neurIST project (www.aneurist.org), and Aneurisk (http://ecm2.mathcs.emory.edu/aneuriskweb/index)^30^, providing access to over 700 cerebral aneurysm geometries extracted from patients in Sheffield, Milan, Geneva, and Barcelona. The dataset comprises 253 cases of ruptured aneurysms and 456 cases of unruptured aneurysms. Figure 1 provides insights into the dataset, presenting information on the distribution of aneurysm locations within the Circle of Willis and the gender distribution of the patients. The dataset shows that the Internal Cerebral Artery (ICA), the Middle Cerebral Artery (MCA), and the Anterior Communicating Artery (ACA) are the most frequent locations for an aneurysm within the Circle of Willis, with most cases belonging to female patients.

**Figure 2:**
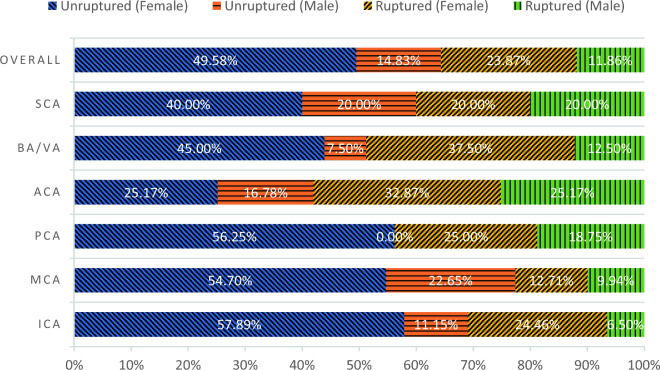
Rupture status scattering based on location and sex.

Figure 2 determines the rupture status based on specific aneurysm locations and biological genders. The figure illustrates the rupture rates associated with different anatomical locations. According to the data presented in Fig. 2, the Anterior Communicating Artery (ACA), the Basilar/Vertebral Artery (BA/VA), and the Posterior Communicating Artery (PCA) are identified as the most precarious locations, exhibiting a rupture rate exceeding 40%, regardless of gender.

### Clinical and morphological parameters

The features considered in our analysis can be divided into two main groups: three clinical parameters, namely age, gender, and aneurysm location in the Circle of Willis, and morphological features that relate to the geometric characteristics of each aneurysm. To fully comprehend the contribution of aneurysm geometry to the rupture status over time, we aimed to include a diverse set of morphological features consisting of 35 distinct parameters. This section outlines 27 morphological parameters, including parameters previously used by other researchers, and eight novel parameters, including neck circumference, ideal roundness, ideal roundness ratio, ideal sphericity, ideal sphericity ratio, outflow number, cumulative outlet diameter, and inlet to outlet(s) ratio, that we aim to introduce and compare with existing parameters to assess their contribution in cerebral aneurysm rupture. Table 1 provides an overview of the parameters that are recognized characteristics of cerebral aneurysms and were drawn from previous literature. While some studies have employed certain parameters in training machine learning models and investigating rupture status, no study has comprehensively considered all of them simultaneously to compare their contributions. Our approach extends beyond the incorporation of previously recognized parameters. We introduce eight new parameters for the first time, intending to synergize them with established features. This approach achieves a deeper and more nuanced understanding of the morphological parameters that cause cerebral aneurysms rupture.

**Table 1:** Definition of morphological parameters.

Parameters	Definition
Dome volume (DV)	Insider volume of the saccular
Dome area (DA)	Outer area of the saccular
Maximum diameter (MD)	Maximum length between two distinct points on the saccular
Central perpendicular height (CPH)	Perpendicular height from the center of the neck
Neck diameter (ND)	Diameter of the neck parallel to blood flow
Neck area (NA)	Area of the neck
Diameter of the parent vessel at the inlet (DPVI)	Diameter of parent vessel before aneurysm
Maximum perpendicular height (MPH)	Maximum perpendicular height from the neck plane
Inflow angle (IMDA)	Angle between the inlet flow and MD
Aneurysm angle (MDNA)	Angle between the neck and MD
Neck angle (INA)	Angle between the inlet flow and neck
Projection length (PL)	Length of the saccular shadow on the parent vessel
Maximum diameter parallel to the neck (MDPN)	Distance between the neck and a parallel cut-plane of the saccular with the largest area
Lateral or bifurcation (LB) status	Location of the aneurysm in the parent vessel
Irregularity (I)	Deviation from the balloon shape
Aspect ratio (AR)	$CPH / ND$
Bottleneck ratio (BR)	$MD / ND$
Neck ratio (NR)	$ND / DPVI$
Size ratio (SR)	$MD / DPVI$
Dome to neck ratio (DNR)	$DA / NA$
Shape factor (SF)	$DA/DV^{2/3}$
Volume to the ostium area (VOA)	$DV/NA$
Ellipticity index* (EI)	$1 - \left( {18\pi } \right)^{\frac{1}{3}} DV_{CH}^{\frac{2}{3}} /DA_{CH}$
Nonsphericity shape index (NSI)	$1 - \left( {18\pi } \right)^{\frac{1}{3}} DV^{\frac{2}{3}} /DA$
Undulation index* (UI)	$1 - DV/DV_{CH}$
Projection ratio (PR)	$MPH/DPVI$
Conicity parameter (CP)	$0.5 - MDPN/DPVI$

*$DV_{CH}$ and $DA_{CH}$ are the volume and outer area of the convex hull of the aneurysm^31,32^.

**Figure 3:**
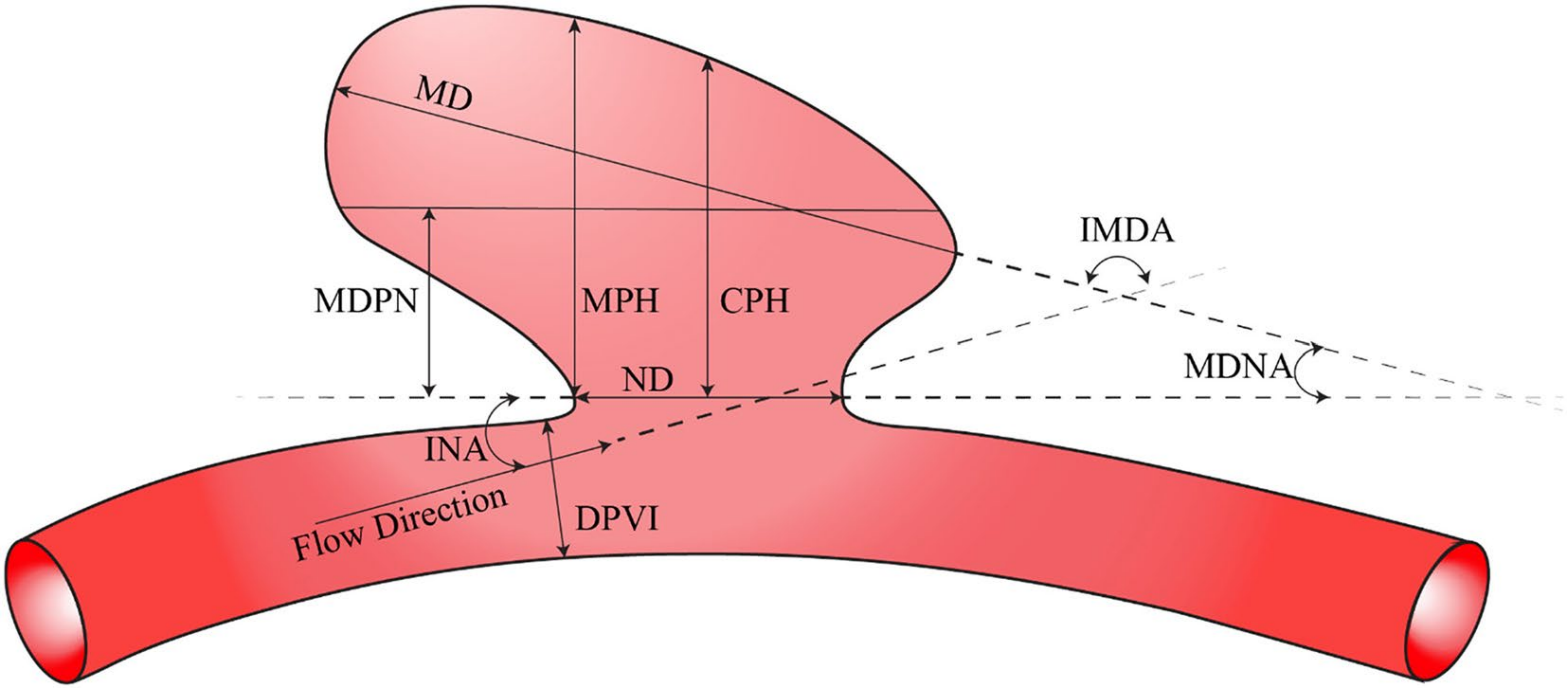
Depiction of some morphological parameters.

The dataset included several parameters that were already measured, and we used the data provided for these parameters. The available parameters include dome volume, dome area, perpendicular height, maximum diameter parallel to the neck, neck diameter, aspect ratio, bottleneck ratio, ellipticity index, non-sphericity shape index, undulations index, and conicity parameter. In addition, we measured the remaining 16 parameters using Materialize 3-matic v13.0, ParaView v5.11.0, and mathematical formulas. This meticulous approach ensures a thorough evaluation of the morphological features that contribute to the rupture status of cerebral aneurysms. Figure 3 presents a schematic for a subset of the parameters mentioned earlier, as outlined in Table 1, which includes the three angles considered in this study.

### Novel morphological parameters

Although many morphological parameters have been introduced for cerebral aneurysms in the past, some of which have been used to train machine learning models to investigate their contributions to the rupture status of aneurysms, there are still parameters that are worth exploring for this purpose. In this study, we examined eight previously unused parameters to investigate their effects on rupture status using machine learning. Definition of these parameters are available in Table 2. To account for the fact that the neck of an aneurysm may not be circular, we measure not only the circumference of the neck but also the radius of a circle with the same area as the neck. This allowed us to introduce another dimensionless parameter called the ideal roundness ratio, which is the ratio of the circumference of the circle with the equivalent area of the neck to the actual perimeter of the neck. This ratio is always less than 1 because, among all 2D shapes with the same area, the circle has the smallest perimeter.

**Table 2:** Definition of new morphological parameters.

New parameters	Definition
Neck circumference (NC)	Perimeter of the neck
Ideal roundness (IR)	$\sqrt {NA/\pi }$
Ideal roundness ratio (IRR)	$2\pi \left( {IR} \right)/NC$
Ideal sphericity (IS)	$$\sqrt[3]{{3\left( {DV} \right)/4\pi }}[3]$$$${{3\left( {DV} \right)/4\pi }}$$
Ideal sphericity ratio (ISR)	$4\pi \left( {IS} \right)^{2} /\left( {DA + NA} \right)$
Outflow number (ON)	Number of outlets from the saccular
Cumulative outlet diameter (COD)	Total diameter of all outlets from the saccular
Inlet to outlet(s) ratio (IOR)	$DPVI/COD$

**Figure 4:**
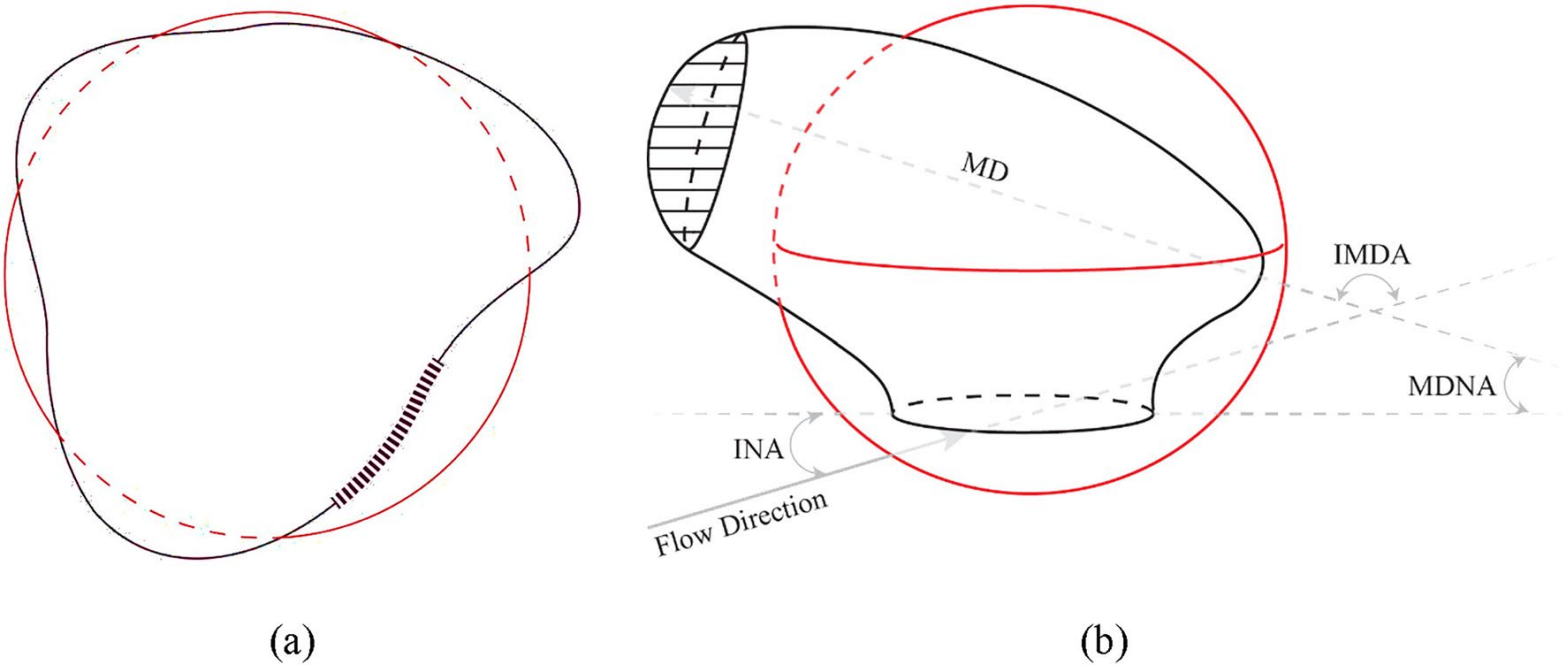
(**a**) IR and IRR, (**b**) IS and ISR (IR, IS, IRR, and ISR—are independent of flow direction and angles).

To better understand the concept of ideal roundness and its ratio, please refer to Fig. 4a. The solid black curve represents the actual neck perimeter, whereas the circle represents a shape with the same area as the neck. The radius of this circle is considered the ideal roundness, and the ideal roundness ratio is the ratio of the circumference of the circle to the perimeter of the neck. The hatched segment along the line illustrates the length disparity between the circumference of the circle and the perimeter of the neck.

Applying a similar strategy to the volume, we introduced two additional parameters, ideal sphericity and ideal sphericity ratio, as illustrated in Fig. 4b. Ideal sphericity refers to the radius of a sphere with the same volume as the saccular section of the aneurysm, whereas the ideal sphericity ratio is the ratio of the outer surface area of the equivalent sphere (with the same volume as the aneurysm) to the real outer area of the aneurysm, encompassing both the saccular outer area and the neck area. In Fig. 4b, the hatched segment illustrates the additional outer area of the saccular section compared with the sphere of the same volume. This ratio must also be less than 1 because, among all geometries with the same volume, the sphere has the smallest outer surface area. Next, we introduced three additional parameters: outflow numbers, cumulative outlet diameter, and inlet-to-outlet(s) ratio. Although lateral aneurysms typically have only one outlet, considering bifurcation and lateral cases simultaneously (with the majority being bifurcation), numerous geometries exhibit multiple outlets based on the location of the aneurysm on the parent artery. The cumulative outlet diameter represents the sum of the diameters of all outlets from the aneurysm, and the inlet-to-outlet(s) ratio is the ratio of the inlet diameter to the total diameter of all outlets.

### Machine learning

The first step in this section is standardization to reduce the impact of outliers. Five supervised models were explored to distinguish between ruptured and unruptured cerebral aneurysms: Support Vector Machine (SVM), k-nearest neighbors (KNN), Random Forest (RF), Extreme Gradient Boosting (XGB), and Multilayer Perceptron (MLP). These models are widely recognized for their effectiveness in classification problems. Notably, MLP operates within the domain of neural networks. We explored various architectures using the grid search method to identify the most suitable hyperparameters for our dataset. This approach to model selection and hyperparameter tuning enhanced the robustness and precision of our predictions for the classification of ruptured and unruptured cerebral aneurysms. Moreover, we performed five-fold cross-validation for all models to minimize the effect of random splitting and improve the reliability of the results. The MLP model used three hidden layers with an adaptive learning rate and identity activation function.

## Results and discussion

In this section, we discuss the outcomes produced by different machine learning models, aiming to compare and determine the most effective model for predicting cerebral aneurysm rupture based on 35 morphological and 3 clinical inputs. The evaluation criteria include accuracy for the train and test datasets, recall, precision, and accuracy for the test dataset and the receiver characteristic operation (ROC) curve. Following these evaluations for each model, we discuss the most significant features identified by the models. We aim to shed light on the correlation between each parameter and the rupture status of cerebral aneurysms. This analysis provides a comprehensive understanding of the influential factors contributing to the accurate prediction of aneurysm rupture.

### Accuracy

**Figure 5:**
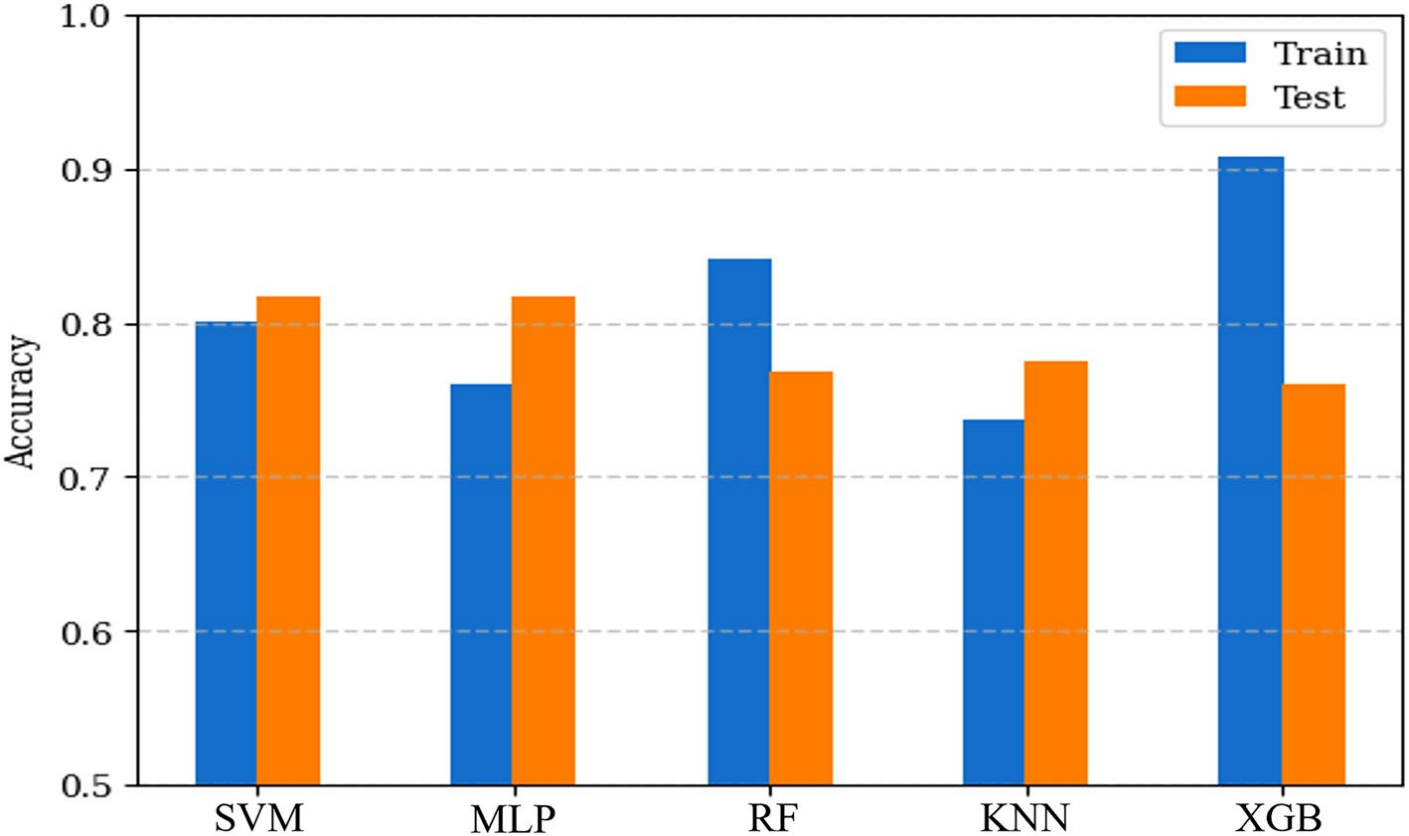
Accuracy of train and test datasets.

The main metric for evaluating model performance and enabling comparisons between different models is accuracy, which is measured on both the train and test datasets. Accuracy is defined as the ratio of correctly predicted cases to all predicted cases. It is important to note that while high accuracy is desirable, achieving 100% accuracy is not optimal, as it may indicate overfitting and a lack of generalization to unseen data. Ideally, the train and test datasets should have similar accuracy, with a recommended maximum difference of 10%. In Fig. 5, we present the accuracy results for all models. It is evident that all models can achieve an accuracy exceeding 0.70. XGB demonstrates the highest accuracy at 0.91, while KNN exhibits the lowest accuracy at 0.74. Assessing the generalizability of the models to new data, both MLP and SVM demonstrated superior performance, achieving an accuracy of 0.82 for the test dataset. This indicates that MLP and SVM outperform the other models in terms of predictive accuracy for unseen data.

### Precision and recall

In addition to accuracy, we included precision and recall as important metrics to comprehensively evaluate model performance. We made this decision due to the sensitivity of the medical data under consideration, emphasizing the importance of timely disease recognition. In simple terms, recall measures the model’s ability to correctly identify the presence of a disease. Recall is defined as the ratio of true positive predictions to the total number of actual positive cases. Similarly, precision reflects the model’s ability to accurately predict positive occurrences. Precision is defined as the ratio of true positive predictions to the total number of predicted positive cases.

**Figure 6:**
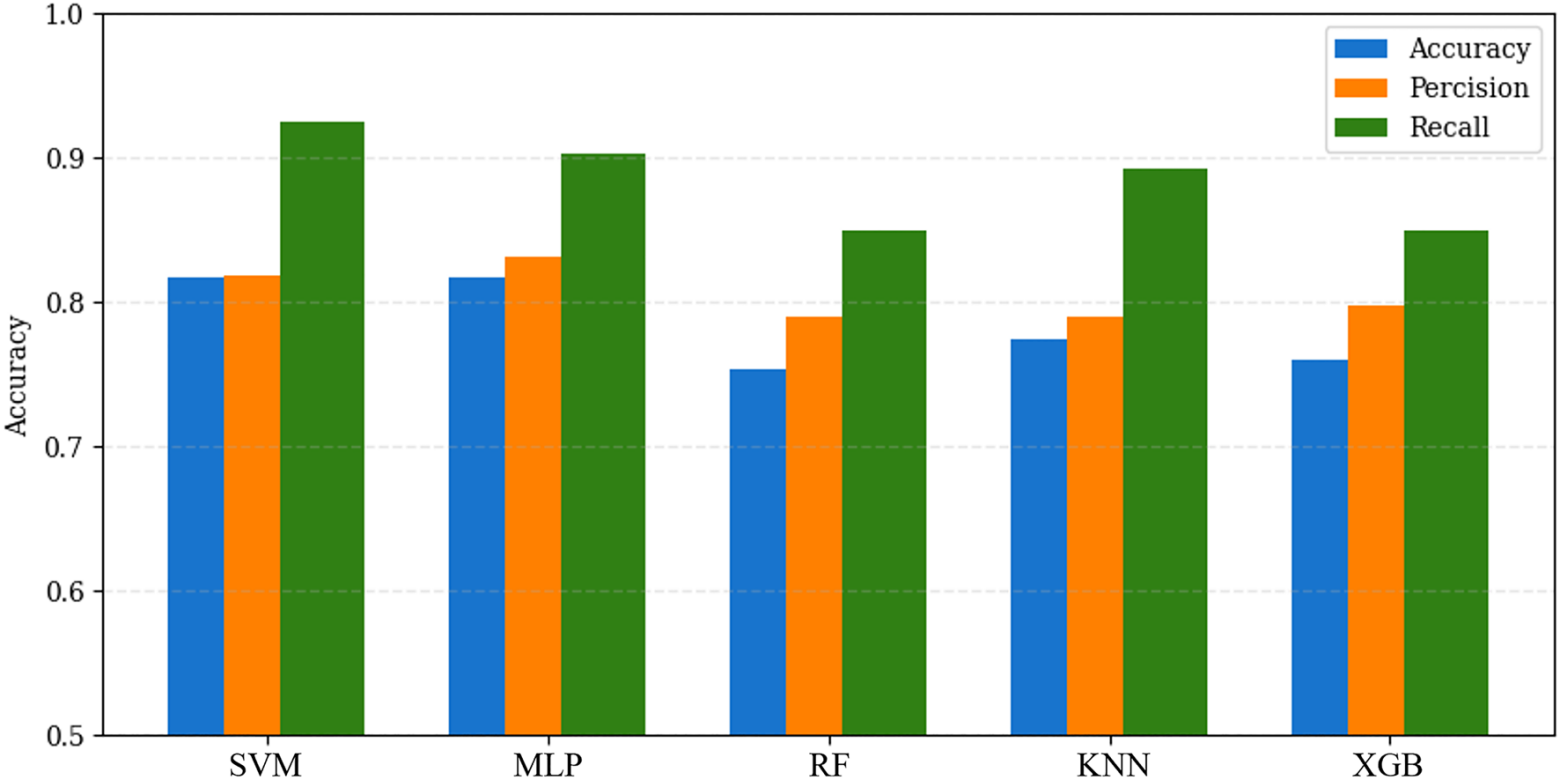
Accuracy, precision, and recall for the test dataset.

In the medical context, recall holds particular significance, but accuracy and precision should not be overlooked, as they collectively contribute to overall model efficacy. Figure 6 presents the evaluation of all three metrics (accuracy, precision, and recall) for the test dataset, with a specific focus on the ruptured class, representing the occurrence scenario in our study. SVM and MLP are the top-performing models once again. The results show that SVM and MLP have high recall rates of 0.92 and 0.90, respectively, in predicting the occurrence of cerebral aneurysm rupture. SVM also has an accuracy and precision of 0.82, whereas MLP has a precision of 0.83 and an accuracy of 0.82. In contrast, RF performed relatively poorly in all three criteria. However, it is noteworthy that even for RF, all performance metrics for the test dataset exceeded 0.75, indicating a high level of predictive capability.

### ROC curve

**Figure 7:**
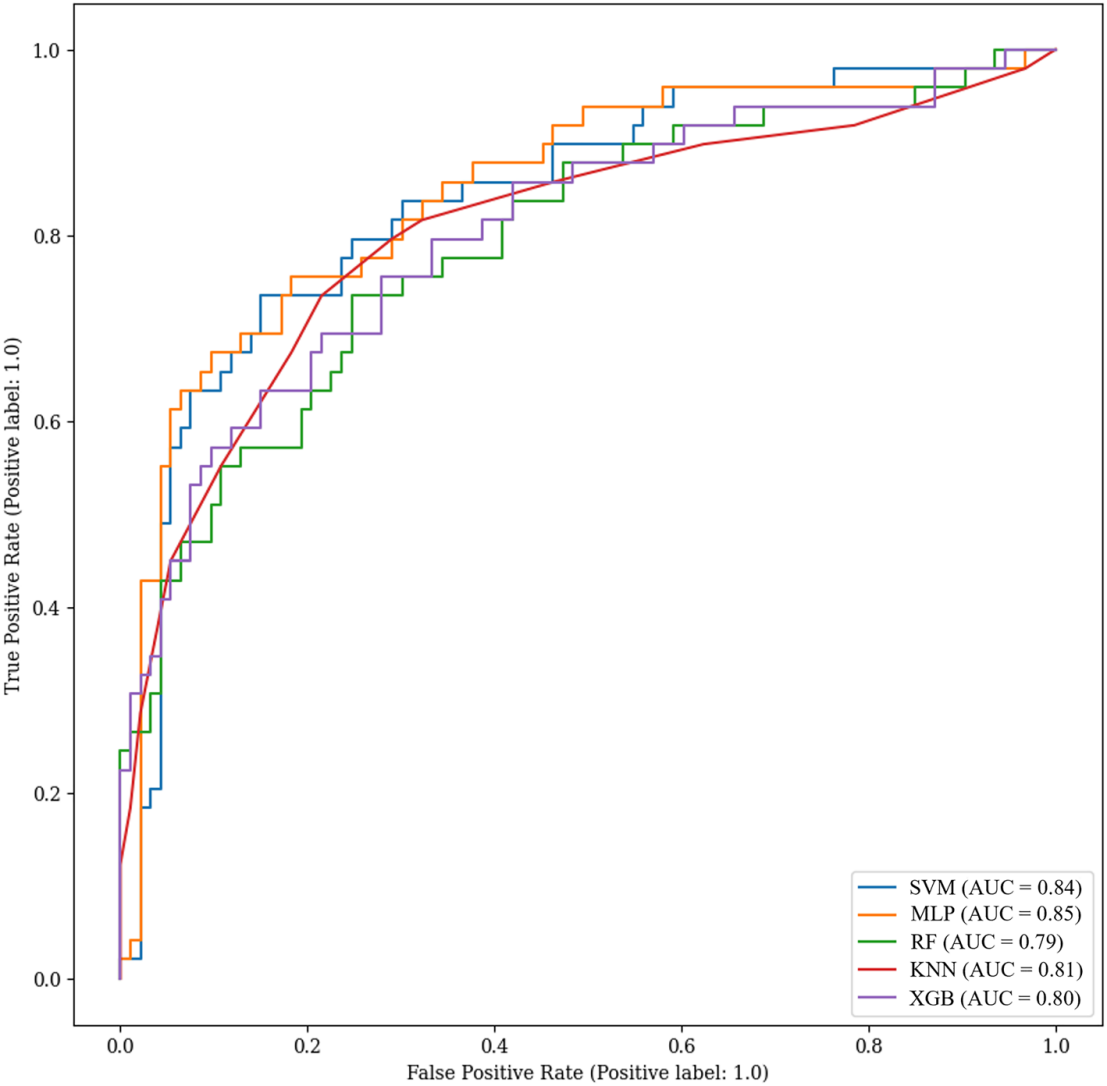
Receiver operating characteristic (ROC) curve for all models.

Another metric used for evaluation is the ROC curve, which illustrates the true positive rate versus the false positive rate. Linear behavior, where the true and false positive rates are equal, represents a random classifier. As the model improves, the curve shifts toward the upper-left point. An ideal model would have a true positive rate of 1 and a false positive rate of 0. The area under the curve (AUC) is a representative measure of the model’s performance, with an AUC of 0.5 indicating a random classifier and an AUC of 1 indicating an ideal classifier. Figure 7 presents the behavior of the ROC curve for each model, along with the corresponding AUC. Based on these criteria, SVM and MLP are the top-performing models engaged in close competition. Their ROC curves exhibit a favorable trajectory, and their AUC values affirm their strong performance. Conversely, RF demonstrates a comparatively poorer performance than the other models. In summary, all models demonstrate highly acceptable performance and scores. Optimizing these models to improve their reliability and effectiveness in predicting cerebral aneurysm rupture represents a valuable endeavor.

### Dominant features

**Figure 8:**
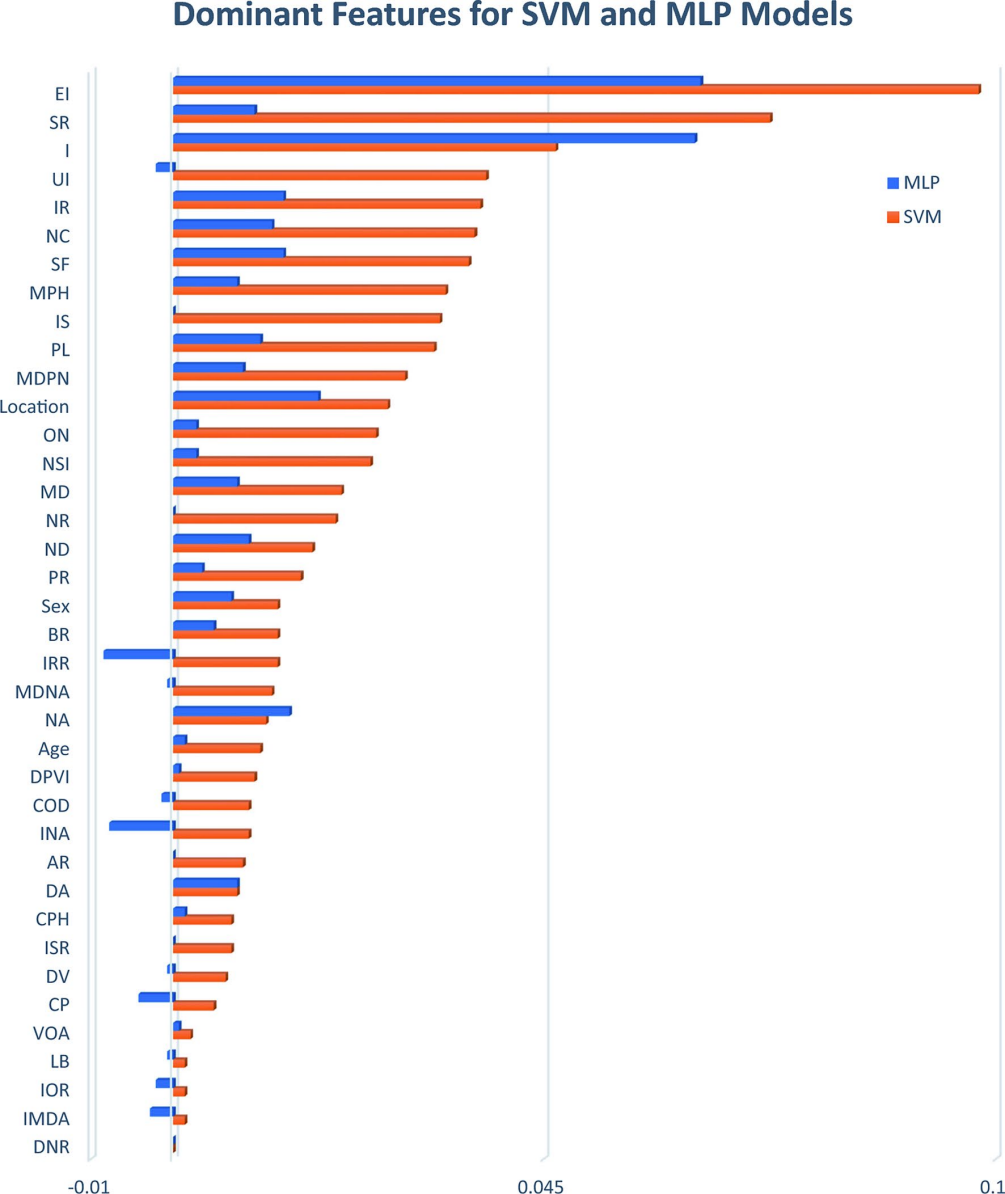
Feature importance for the two top-performing models.

Given that each machine learning model employs a unique set of algorithms and mathematical relations, a difference in the weight assigned to each parameter for the final classification decision is expected. Figure 8 displays the weights of each parameter for the two top-performing models in this study. The SVM model identifies the first five important features as EI (Ellipticity Index), SR (Size Ratio), I (Irregularity), UI (Undulation Index), and IR (Ideal Roundness), a new parameter introduced in this study. The MLP model, on the other hand, prioritizes EI, I, Location, NA (Neck Area), and IR, with IR once again demonstrating a significant impact.

Other novel parameters introduced in this study include NC, IS, ON, IRR, COD, ISR, and IOR, which occupy positions 6, 9, 13, 19, 27, 30, and 36, respectively, for SVM. For the MLP model, the order of these new parameters is IR (5), NC (7), ON (18), IS (24), ISR (27), COD (32), IOR (34), and IRR (38). Notably, some parameters for the MLP model exhibit negative values, indicating an inverse effect on the model’s prediction and an inverse correlation with the output. It is important to acknowledge that this pattern may vary depending on the architecture used for the MLP model.

One potential question that may arise in this study is whether bifurcation aneurysms are more prone to rupture than lateral aneurysms, based on physicians’ experience. However, our study does not show a significant contribution from this factor. This discrepancy does not imply that the bifurcation and lateral status are insignificant. Instead, it highlights that when other features are considered alongside this parameter, there is a stronger correlation among other parameters than with this specific one. Essentially, by expanding our input variables and making decisions based on more comprehensive information, we uncover the significance of parameters that may not have been previously considered. Thanks to modern machine learning models, it is now possible to compare several parameters simultaneously and discern the contribution of each in relation to others. This approach allows for more reliable decision-making by considering a broader set of factors and better understanding the complex interplay of variables that contribute to the prediction of cerebral aneurysm rupture.

We now undertake a brief comparison between prior research and the current study, focusing specifically on the testing datasets utilized across all studies. To facilitate this analysis, we direct attention to Table 3 which presents the outcomes of six comparable studies alongside those of our own investigation. As previously indicated, we endeavored to incorporate a comprehensive array of morphological parameters to ensure the robustness of our findings.

**Table 3:** Comparison between the current study and similar previous studies.

Authors	Number of aneurysms	Number of morphological parameters	Important morphological parameters	Number of ML algorithms	Best ML model and its scores
Tanioka et al.^23^	226	10	Projection ratio size ratio aspect ratio	1	RF (Accuracy = 0.77)
Silve et al.^33^	845	3	Aneurysm size	3	RF (AUC = 0.81)
Zhu et al.^34^	668	4	N.A	1	SVM (AUC = 0.62)
Ou et al.^24^	374	6	Size ratio	4	XGBoost (AUC = 0.88) (Accuracy = 0.84)
Amigo et al.^35^	71	11	Size ratio	8	RF (AUC = 0.82) (Accuracy = 0.68)
Detmer et al.^25^	1631	25	Surface curvature Aneurysm width Volume-to-ostium ratio	6	MLP (AUC = 0.826) (Accuracy = 0.78)
Current Study	708	35	Ellipticity index size ratio irregularity	5	SVM (AUC = 0.84) (Accuracy = 0.82) (Precision = 0.82) (Recall = 0.92)

As the scope of parameters considered expands, it is anticipated that there will be shifts in the relative importance assigned to each parameter. Furthermore, increasing the size of the dataset can enhance the reliability of the results. Among the parameters of significance, the size ratio emerges as a recurrent focal point, underscoring its inherent importance in assessing the risk of rupture. Once more, we underscore the significance of the recall score, given the sensitivity inherent in medical data. Notably, our study achieves an outstanding recall score, a metric unfortunately absents from prior works, thus limiting direct comparison.

## Conclusion

This study used data from more than 700 patients diagnosed with cerebral aneurysms to extract a comprehensive set of morphological parameters from the geometries. We combined this information with clinical features to train machine learning models to predict the occurrence of rupture in advance. The consideration encompassed 3 clinical and 35 morphological features, including 8 novel parameters introduced in this study that were not previously explored in any other research. This study investigated various morphological parameters to gain a better understanding of the factors associated with cerebral aneurysm rupture. We used five distinct machine learning algorithms to establish connections between the available features and the incidence of aneurysm rupture. Two of our models, SVM and MLP, demonstrated the ability to predict aneurysm rupture with an accuracy of 0.82 for the test dataset, demonstrating their robust performance on unseen data. Furthermore, we achieved remarkable recall scores of 0.92 and 0.90 for SVM and MLP models, respectively, underscoring their exceptional capability to correctly predict real patient outcomes. The features that most strongly correlated with the rupture status of aneurysms varied between models. The ellipticity index, size ratio, and irregularity were the most influential features for the SVM model, while the MLP model identified the ellipticity index, irregularity, and location as pivotal. Notably, ideal roundness, a newly introduced parameter, ranked fifth among all 38 parameters, indicating a significant correlation with rupture status. Furthermore, this study introduces other novel parameters, such as neck circumference, number of outlets, and ideal sphericity, which have been shown to be important contributors. Neck circumference ranked sixth as an important feature in SVM and seventh in MLP, further emphasizing the value of exploring previously unconsidered features. In conclusion, our study provides valuable insights into the prediction of cerebral aneurysm rupture, demonstrating the effectiveness of machine learning algorithms in integrating a diverse set of features for accurate prediction. The identification of key parameters and the introduction of novel variables contribute to a deeper understanding of the factors influencing cerebral aneurysm rupture. The next step in continuing this study could be to explore a comprehensive set of hemodynamic parameters to investigate the fluid mechanics effects of blood flow, that contribute to the rupture of an aneurysm. Additionally, combining morphological and hemodynamic parameters, optimizing current classifiers, and considering more machine learning algorithms can be considered.

## Data Availability

The human data used in this study are publicly available at zenodo.org, providing access to over 700 cerebral aneurysm geometries from patients in Sheffield, Milan, Geneva and Barcelona. The data sets analyzed in the current study are available from the corresponding author upon reasonable request.
