# Author Correction: A comprehensive investigation of morphological features responsible for cerebral aneurysm rupture using machine learning

**DOI:** 10.1038/s41598-025-12368-x

**Published:** 2025-08-20

**Authors:** Mostafa Zakeri, Amirhossein Atef, Mohammad Aziznia, Azadeh Jafari

**Affiliations:** 1 CNNFM Lab, School of Mechanical Engineering, College of Engineering, University of Tehran, 1450 Kargar St. N., Tehran, 14399-57131 Iran; 2 Present Address: STRETCH Lab, Department of Biomedical Engineering and Mechanics, Virginia Tech, 330A Kelly Hall, 325 Stanger Street, Blacksburg, VA 24061 USA

Correction to: *Scientific Reports* 10.1038/s41598-024-66840-1, published online 09 July 2024

The original version of the Article contained errors.

Firstly, in the Methodology section, under the subheading “Study population”, the AneuX morphology database was not declared and cited in accordance with the terms of use. Consequently,

“The dataset used in this study was graciously provided by zenodo.org^33^, which provided access to over 700 cerebral aneurysm geometries extracted from patients in Sheffield, Milan, Geneva, and Barcelona.”

now reads

“The dataset used in this study was graciously provided by the AneuX morphology database, an open-access, multi-centric database combining data from three European projects: AneuX project (www.aneux.ch), @neurIST project (www.aneurist.org), and Aneurisk (http://ecm2.mathcs.emory.edu/aneuriskweb/index)^30^, providing access to over 700 cerebral aneurysm geometries extracted from patients in Sheffield, Milan, Geneva, and Barcelona.”

and Reference 33 listed below:

33. N. Juchler, Bijlenga, Philippe, & Hirsch, Sven., "AneuX morphology database (v1.0)," 2022.

was replaced with:

30. N. Juchler, S. Schilling, P. Bijlenga, V. Kurtcuoglu, and S. Hirsch, “Shape trumps size: image-based morphological analysis reveals that the 3D shape discriminates intracranial aneurysm disease status better than aneurysm size,” *Front. Neurol.* **13**, 809391 (2022).

As a result of the changes, the References have been renumbered.

Secondly, in the Methodology section, under the subheading “Machine learning”, model cross-validation was omitted. As a result,

“This approach to model selection and hyperparameter tuning enhanced the robustness and precision of our predictions for the classification of ruptured and unruptured cerebral aneurysms. The MLP model used three hidden layers with an adaptive learning rate and identity activation function.”

now reads

“This approach to model selection and hyperparameter tuning enhanced the robustness and precision of our predictions for the classification of ruptured and unruptured cerebral aneurysms. Moreover, we performed five-fold cross-validation for all models to minimize the effect of random splitting and improve the reliability of the results. The MLP model used three hidden layers with an adaptive learning rate and identity activation function.”

Thirdly, in the Results and discussion section, under the subheading “Dominant features”, the text was updated to reflect more commonly accepted terminology. The subheading title has been revised to “Feature importance” and as a result,

“The SVM model identifies the first five dominant features as EI (Ellipticity Index), SR (Size Ratio), I (Irregularity), UI (Undulation Index), and IR (Ideal Roundness), a new parameter introduced in this study.”

now reads

“The SVM model identifies the first five important features as EI (Ellipticity Index), SR (Size Ratio), I (Irregularity), UI (Undulation Index), and IR (Ideal Roundness), a new parameter introduced in this study.”

and

“We now undertake a brief comparison between prior research and the current study, focusing specifically on the testing datasets used across all studies. To facilitate this analysis, we refer to which presents the outcomes of six comparable studies alongside those of our own investigation. As previously indicated, we endeavored to incorporate a comprehensive array of morphological parameters to ensure the robustness of our findings.

As the scope of parameters considered expands, shifts in the relative importance assigned to each parameter are anticipated. Furthermore, increasing the size of the dataset can enhance the reliability of the results. Among the parameters of significance, the size ratio emerges as a recurrent focal point, underscoring its inherent importance in assessing the risk of rupture. Once more, we underscore the significance of the recall score, given the sensitivity inherent in medical data. It is noteworthy that our study achieves an outstanding recall score, a metric that is unfortunately absent from prior studies, thus limiting direct comparison.

Table 3, which presents the outcomes of six comparable studies alongside those of our own investigation. As previously indicated, we endeavored to incorporate a comprehensive array of morphological parameters to ensure the robustness of our findings.

As the scope of parameters considered expands, shifts in the relative importance assigned to each parameter are anticipated. Furthermore, increasing the size of the dataset can enhance the reliability of the results. Among the parameters of significance, the size ratio emerges as a recurrent focal point, underscoring its inherent importance in assessing the risk of rupture. Once more, we underscore the significance of the recall score, given the sensitivity inherent in medical data. It is noteworthy that our study achieves an outstanding recall score, a metric that is unfortunately absent from prior studies, thus limiting direct comparison.”

now reads

“We now undertake a brief comparison between prior research and the current study, focusing specifically on the testing datasets utilized across all studies. To facilitate this analysis, we direct attention to Table 3 which presents the outcomes of six comparable studies alongside those of our own investigation. As previously indicated, we endeavored to incorporate a comprehensive array of morphological parameters to ensure the robustness of our findings.

As the scope of parameters considered expands, it is anticipated that there will be shifts in the relative importance assigned to each parameter. Furthermore, increasing the size of the dataset can enhance the reliability of the results. Among the parameters of significance, the size ratio emerges as a recurrent focal point, underscoring its inherent importance in assessing the risk of rupture. Once more, we underscore the significance of the recall score, given the sensitivity inherent in medical data. Notably, our study achieves an outstanding recall score, a metric unfortunately absents from prior works, thus limiting direct comparison.”

Additionally, in the “Conclusion” section,

“Neck circumference ranked sixth as a dominant feature in SVM and seventh in MLP, further emphasizing the value of exploring previously unconsidered features.”

now reads

“Neck circumference ranked sixth as an important feature in SVM and seventh in MLP, further emphasizing the value of exploring previously unconsidered features.”

Furthermore, Figure 8 and the respective legend were updated. The original Figure 8 and accompanying legend appear below.

**Fig. 8:**
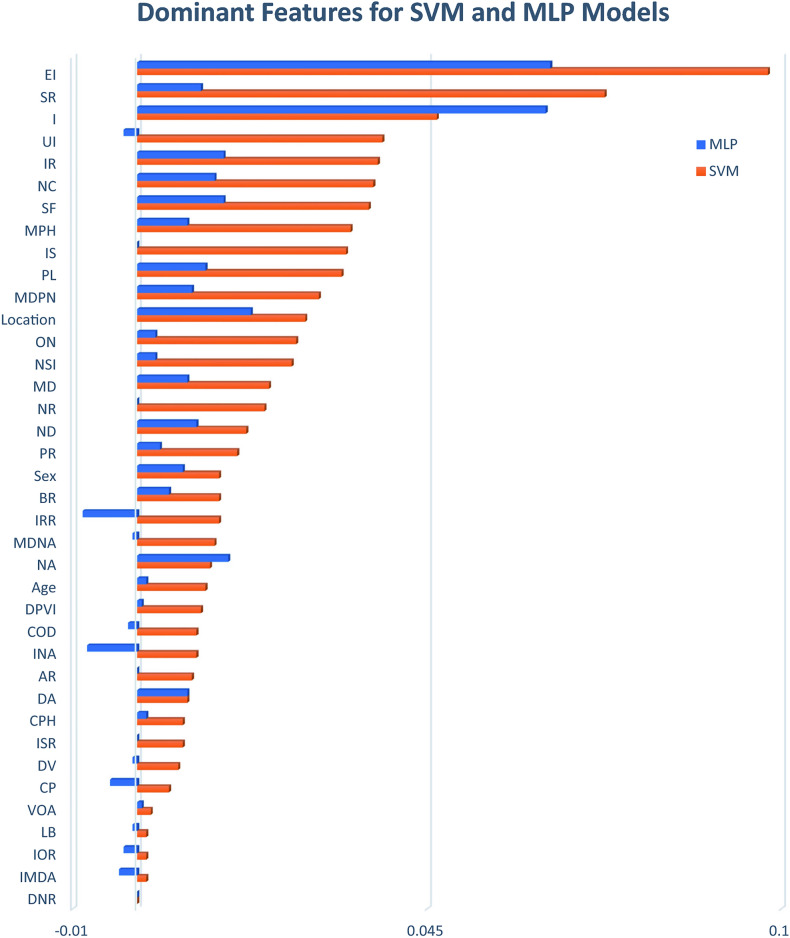
Dominant Features for the two top-performing models.

The original Article has been corrected.

